# Criteria for determining the need for surgical treatment of tricuspid regurgitation during mitral valve replacement

**DOI:** 10.1186/1749-8090-7-27

**Published:** 2012-03-25

**Authors:** Jigang He, Zhenya Shen, Yunsheng Yu, Haoyue Huang, Wenxue Ye, Yinglong Ding, Shaolei Yang

**Affiliations:** 1 Department of Cardiovascular Surgery, First Affiliated Hospital of Soochow University, Suzhou, Jiang Su 215006, People's Republic of China

**Keywords:** Surgical intervention, Mitral valve disease, Tricuspid valve regurgitation, Criteria

## Abstract

**Background:**

Tricuspid regurgitation (TR) is common in patients with mitral valve disease; however, there are no straightforward, rapidly determinably criteria available for deciding whether TR repair should be performed during mitral valve replacement. The aim of our retrospective study was to identify a simple and fast criterion for determining whether TR repair should be performed in patients undergoing mitral valve replacement.

**Methods:**

We reviewed the records of patients who underwent mitral valve replacement with or without (control) TR repair (DeVega or Kay procedure) from January 2005 to December 2008. Preoperative and 2-year postoperative echocardiographic measurements included right ventricular and atrial diameter, interventricular septum size, TR severity, ejection fraction, and pulmonary artery pressure.

**Results:**

A total of 89 patients were included (control, n = 50; DeVega, n = 27; Kay, n = 12). Demographic and clinical characteristics were similar between groups. Cardiac variables were similar between the DeVega and Kay groups. Right atrium and ventricular diameter and ejection fraction were significantly decreased postoperatively both in the control and operation (DeVega + Kay) group (*P *< 0.05). Pulmonary artery pressure was significantly decreased postoperatively in-operation groups (*P *< 0.05). Our findings indicate that surgical intervention for TR should be considered during mitral valve replacement if any of the following preoperative criteria are met: right atrial transverse diameter > 57 mm; right ventricular end-diastolic diameter > 55 mm; pulmonary artery pressure > 58 mmHg.

**Conclusions:**

Our findings suggest echocardiography may be used as a rapid and simple means of determining which patients require TR repair during mitral valve replacement.

## Background

Approximately 30% of patients with mitral stenosis have at least moderate tricuspid regurgitation (TR) [1,2]. Further, findings from several studies have demonstrated that 23% to 37% of patients develop serious TR after mitral valve replacement for rheumatic heart disease [3,4]. Matsuyama et al. [5] have also reported that 37% of patients who had Grade II TR before surgery developed > moderate TR after mitral valve replacement. This percentage is even higher for patients who receive surgical intervention for ischemic mitral valve disease, with up to 74% developing moderate to severe TR within 2 years of surgery [6,7]. The high prevalence of TR after mitral valve replacement is of clinical interest given that TR can result in heart failure, cardiac cachexia, and a general poor prognosis.

Over the last decade, a number of studies have examined the value of correcting TR in patients undergoing mitral valve repair [8-11]. Dreyfus et al. [12] found that both hospital mortality and actuarial survival were increased, although not significantly so, in patients who underwent mitral valve replacement and tricuspid annuloplasty compared with patients who underwent mitral valve replacement alone. Further, the proportion of patients with > Grade II TR was significantly lower (2%) after mitral valve replacement and tricuspid annuloplasty compared with mitral valve replacement alone (48%). In another study, De Bonis et al. [13] found that 12% of patients with TR caused by mitral regurgitation who underwent mitral valve repair and tricuspid annuloplasty experienced ≥ Grade III TR 2 years after surgery. Interestingly, Koukoui et al. [14] reported that TR was uncommon in patients with mitral valve prolapse after surgery in patients who did not have TR before treatment.

Clearly, some patients are more likely to benefit from tricuspid annuloplasty during mitral valve replacement. Both the American College of Cardiology/American Heart Association (ACC/AHA) [15] and the European Society of Cardiology (ESC) [16] recommend (Class I) performing mitral valve surgery with tricuspid annuloplasty for patients with severe TR. The ESC also recommends (Class IIa) simultaneous tricuspid annuloplasty for patients with a tricuspid valve diameter > 40 mm or moderate TR [16]. In contrast, the ACC/AHA recommends (Class IIb) simultaneous tricuspid annuloplasty for patients with moderate or mild TR [15]. The ESC also recommends (Class IIa) tricuspid annuloplasty before right heart failure and severe pulmonary hypertension if there are isolated symptoms of TR after left heart valve surgery [16]. Antunes and Barlow [8] have suggested that tricuspid annuloplasty should be performed for patients with greater than mild TR and at least one of the following: rheumatic valve disease; tricuspid valve annulus diameter > 21 mm/m^2^; expansion of the right heart chamber; expansion of the inferior vena cava; or right ventricular overload.

The purpose of this retrospective study was to identify a simple and fast criterion for determining whether TR repair should be performed in patients undergoing mitral valve replacement.

## Methods

### Patients

The records of 89 consecutive patients who underwent mitral valve replacement surgery from January 2005 to December 2008 and completed 2 years of follow-up were retrospectively reviewed. Of the 89 patients, 39 also underwent tricuspid valve surgery for the treatment of TR (n = 27, DeVega procedure; n = 12, Kay procedure). The 50 patients who did not undergo tricuspid valve surgery served as the control group. Patients who were > 65 years of age received a biological valve for mitral valve replacement, whereas patients who were < 65 years of received a mechanical valve for mitral valve replacement. Inclusion criteria: mitral valve disease with necessary replacement; tricuspid valve disease with functional changes; mitral valve disease combined with tricuspid valve disease. Exclusion criteria: combined with aortic valve disease or other cardiac diseases (e.g. coronary heart disease); tricuspid valve autologous diseases (e.g. caused by rheumatic diseases etc.); mitral valve disease without necessary replacement. This study was approved by Ethics Committee of The First Affiliated Hospital of Soochow University.

### Surgery

The requirement for tricuspid valve surgery was determined with reference to the severity of TR. The severity of TR was determined intraoperatively using the water test, performed after mitral valve replacement and cardioversion. Mild TR was indicated by the right ventricle remaining fully filled after a cardiac cycle; moderate TR was indicated by the right ventricle remaining ≥ 50% filled after a cardiac cycle; and severe TR was indicated by 0% right ventricular filling after a cardiac cycle. If TR was mild, surgical intervention was not performed (these patients were included in the control group). Patients with moderate TR underwent Kay annuloplasty and patients with moderate to severe or severe TR underwent DeVega annuloplasty. In brief, for Kay annuloplasty 1-2 mattress sutures with double-headed noninvasive stitches were placed along the junction between the anterior mitral valve leaflet and the posterior mitral valve leaflet, and at the posterior mitral valve ring. Spacer blocks were applied for both insertion and withdrawal of the needle and the valve annulus was shortened after ligation. For DeVega annuloplasty, a double layer cross-over continuous suture with double-headed noninvasive stitches along the valve rings of the anterior leaflet and the posterior leaflet was placed. Spacer blocks were applied for both insertion and withdrawal of the needle, and the stitches were tightened and ligated to shorten the dilated posterior valve rings at the base of the posterior and anterior leaflets. All surgeries were performed by the same surgeon.

### Measurements

Preoperatively, all patients underwent echocardiographic examinations. The apical 4-chamber view was used to determine the diameter of the right ventricles and atria, the size of the interventricular septum, the degree of TR, and ejection fraction. Pulmonary artery pressure was also assessed. All examinations were performed by the same radiologist. The degree of TR was evaluated using the regurgitation jet area method [17]. The maximum regurgitation area was determined from cross-sectional images, and the ratio between this area and the area of the right atrium was calculated. The severity of TR was defined by the following ratios: < 20% = mild; 20-40% = moderate; > 40% = severe. All patients were followed up for a minimum of 2 years. The same radiologist repeated the echocardiographic examinations 2 years after surgery to determine postoperative changes.

### Statistical analysis

Demographic data are presented as mean ± standard deviation (SD) for age and number (percentage) for categorical variables. Data were compared between groups by one-way analysis of variance for age and Fisher's exact test for other categorical variables. Cardiac variables (including pulmonary artery pressure) are summarized as mean ± SD. Between group comparisons (control vs operation; De Vega vs Kay) and preoperative vs postoperative comparisons were made using two-sample *t*-test. Analysis of covariance was performed to compare cardiac variables among the 3 groups postoperatively if the variables were imbalanced preoperatively. The ordinal data, TR severity, are summarized as number (%) and were compared by Kruskal-Wallis test (among all 3 groups) with a post-hoc pair-wise comparison, Mann-Whitney *U *test (De Vega vs Kay groups). All statistical assessments were two-tailed. Statistical significance was indicated by *P *< 0.05. An adjusted statistical significance level (*P *< 0.0167 [0.05/3]) was used for post-hoc pair-wise comparisons. Statistical analyses were performed using SPSS 15.0 statistical software (SPSS Inc, Chicago, IL).

## Results

Patient demographics were similar between groups (Table 1). Patients were generally aged in their late 40's/early 50's and were predominantly (≥ 60%) female. Most (52-75%) patients had a history of rheumatic heart disease or mitral insufficiency (25-44%). There were no significant between group differences in New York Heart Association functional class before surgery or in the type of bicuspid valve replacement.

**Table 1:** Patient demographic data

Variables	Control group (n = 50)	DeVega group (n = 27)	Kay group (n = 12)	*P*
Age (years)	53.0 ± 13.6	49.8 ± 10.9	49.7 ± 15.9	0.511

Sex				0.327

Male	20 (40)	9 (33)	2 (17)	

Female	30 (60)	18 (67)	10 (83)	

Medical history				0.496

Rheumatic heart disease	26 (52)	18 (67)	9 (75)	

Mitral insufficiency	22 (44)	9 (33)	3 (25)	

Coronary heart disease	2 (4)	0 (0)	0 (00)	

Heart function before surgery (NYHA class)				0.317

I	20 (40)	9 (33)	4 (33)	

II	22 (44)	14 (52)	3 (25)	

III	8 (16)	4 (15)	5 (42)	

Bicuspid valve replacement				0.167

Mechanical prosthesis	33 (66)	23 (85)	8 (67)	

Biovalve	17 (34)	4 (15)	4 (33)	

NYHA, New York Heart Association.

Continuous data are presented as mean ± standard deviation and were compared by one-way analysis of variance. Categorical data are presented as number (percentage) and were compared by Fisher's exact test.

Table 2 summarizes the pre- and postoperative cardiac data for the control and operation (DeVega and Kay) groups. For both groups, right atrium inner diameter, right ventricle inner diameter, and ejection fraction were significantly decreased 2 years after surgery (all *P *< 0.05). Pulmonary artery pressure was significantly decreased 2 years after surgery in the operation group only (*P *< 0.05).

**Table 2:** Pre- and postoperative cardiac data for the control and operation groups

Variable	Control group (n = 50)	**Operation group**^**a**^ (n = 39)
Right atrium inner diameter (mm)		

Preoperative	38.92 ± 3.66	57.21 ± 3.24

2-years postoperative	38.10 ± 3.89†	48.64 ± 3.27†

Right ventricle inner diameter (mm)		

Preoperative	39.12 ± 3.46	54.95 ± 3.19

2-years postoperative	37.10 ± 3.57†	46.18 ± 3.48†

Interventricular septum thickness (mm)		

Preoperative	9.22 ± 1.66	8.74 ± 1.27

2-years postoperative	9.16 ± 2.00	8.82 ± 1.07

Pulmonary artery pressure (mmHg)		

Preoperative	32.14 ± 1.99	57.92 ± 2.91

2-years postoperative	31.96 ± 2.40	31.46 ± 2.83†

Ejection fraction (%)		

Preoperative	0.63 ± 0.09	0.63 ± 0.11

2-years postoperative	0.59 ± 0.09†	0.56 ± 0.10†

^a ^The operation group included patients who underwent DeVega and Kay procedures.

Data are presented as mean ± SD.

† *P *< 0.05, statistically significant difference between preoperative and postoperative values (paired *t*-test).

Table 3 summarizes the pre- and postoperative cardiac data for the DeVega and Kay operation groups. There were no significant between group differences for any of the variables assessed. For both groups, right atrium inner diameter, right ventricle inner diameter, and pulmonary artery pressure were significantly decreased 2 years after surgery (all *P *< 0.05). Ejection fraction was significantly decreased 2 years after surgery in the DeVega operation group only (*P *< 0.05).

**Table 3:** Pre- and postoperative cardiac data for the De Vega and Kay groups

Variable	DeVega group (n = 27)	Kay group (n = 12)	*P*
Right atrium inner diameter (mm)			

Preoperative	57.33 ± 3.08	56.92 ± 3.70	0.716

2-years postoperative	49.11 ± 2.99†	47.58 ± 3.73†	0.737

Right ventricle inner diameter (mm)			

Preoperative	54.96 ± 3.01	54.92 ± 3.70	0.967

2-years postoperative	46.07 ± 3.40†	46.42 ± 3.80†	0.781

Interventricular septum thickness (mm)			

Preoperative	8.93 ± 1.30	8.33 ± 1.16	0.183

2-years postoperative	8.85 ± 1.06	8.75 ± 1.14	0.788

Pulmonary artery pressure (mmHg)			

Preoperative	57.41 ± 2.19	59.08 ± 3.97	0.191

2-years postoperative	31.33 ± 3.26†	31.75 ± 1.55†	0.591

Ejection fraction (%)			

Preoperative	0.63 ± 0.11	0.62 ± 0.10	0.803

2-years postoperative	0.56 ± 0.10†	0.59 ± 0.09	0.452

Data are presented as mean ± SD.

† *P *< 0.05, statistically significant difference between preoperative and postoperative values (paired *t*-test).

Table 4 summarizes TR severity for each group, both pre- and postoperatively. There was a significant difference in TR severity preoperatively (*P *< 0.001). All patients in the control had minor TR, whereas all patients in the DeVega and Kay groups had moderate or severe TR. There was no significant difference in TR severity postoperatively. The vast majority (≥ 83%) of patients in each group had minor TR postoperatively.

**Table 4:** Comparison of tricuspid regurgitation severity

Severity of tricuspid regurgitation	Control group (n = 50)	DeVega group (n = 27)	Kay group (n = 12)	*P*
Preoperative				< 0.001*

Minor	50 (100)	0 (0)	0 (0)	

Moderate	0 (0)	10 (37)	11 (92)	

Severe	0 (0)	17 (63)	1 (8)	

Postoperative				0.736

Minor	45 (90)	25 (93)	10 (83)	

Moderate	4 (8)	0 (0)	2 (17)	

Severe	1 (2)	2 (7)	0 (0)	

Data are presented as number (percentage). Difference among three groups was compared using Kruskal-Wallis test; Difference between two groups (post-hoc pair-wise comparisons) was compared using Mann-Whitney *U *test.

*P < 0.05, statistically significant difference among three groups (Kruskal-Wallis test).

## Discussion

To date, there is no quantitative index available for determining whether surgery for TR is necessary in patients undergoing mitral valve replacement. In the present study, we performed echocardiographic assessments to examine changes in cardiac parameters before and after mitral valve replacement with or without TR repair and to identify criteria for determining the need for TR surgery during mitral valve replacement. Of note, patients who underwent mitral valve replacement with concurrent tricuspid valve surgery for TR (both the DeVega and Kay procedure) had marked postoperative reductions in TR severity. Few patients in the control group (mild TR and mitral valve replacement alone) experienced an increase in TR severity after surgery. Our preoperative echocardiographic findings suggest that surgical intervention for TR is not required for patients with a preoperative right atrial transverse diameter < 40 mm. If this diameter is > 55 mm, surgical intervention is warranted, whereas if the diameter is between 40 and 55 mm, no definitive determination can be made. Surgical intervention for TR is also not warranted for patients with a preoperative right ventricular end-diastolic diameter < 40 mm, but is warranted if this diameter is > 53 mm. If the preoperative right ventricular end-diastolic diameter is between 40 and 53 mm, no definitive determination can be made. Our findings also suggest that surgical intervention for TR is not warranted for patients with a preoperative pulmonary artery pressure < 33 mmHg, but is warranted if this pressure is > 57 mmHg. If the preoperative pulmonary artery pressure is between 33 and 57 mmHg, no definitive determination can be made. For patients with right ventricular end diastolic diameter, right atrial transverse diameter, and pulmonary artery pressure values between the high and low limits, other factors such as age and the cause TR should be considered when evaluating the need for tricuspid valve surgery.

Expansion of the tricuspid annulus is an important factor affecting TR. A normal tricuspid annulus is shaped like saddle, with the highest point located at the junction between the anterior leaflet and the posterior leaflet. However, with increasing TR severity, the tricuspid annulus may expand, becoming flat and round [6,18,19]. Antunes and Barlow reported that during the course of rheumatic disease, rheumatoid process is directly related to TR, which weakens the annulus and causes its expansion [8]. The diameter of a normal tricuspid annulus (2.8 ± 0.5 cm) can be obtained by echocardiography using an apical 4-chamber view and measuring the distance between the base of the septal leaflet and that of the posterior leaflet [20]. Sugimoto et al. [21] reported that there was an excellent correlation between the diameter of the tricuspid valve and the volume of TR. Accordingly, current tricuspid valvuloplasty is mainly focused on annuloplasty and includes the DeVega procedure, the Kay procedure, and procedures using annuloplasty rings (eg, Carpentier ring). In the current study, patients with moderate TR underwent Kay annuloplasty and patients with moderate to severe or severe TR underwent DeVega annuloplasty. Of note, both procedures were equally effective, with low rates of medium and severe TR found 2 years after surgery.

The primary limitations of this study are the relatively small sample size and the short length of follow-up. Of note, there were only 12 patients in the Kay operative group. A larger scale study, with an increased number of patients and a longer duration of follow-up is needed to confirm the findings reported herein.

## Conclusion

In conclusion, we suggest that echocardiography can be employed to determine whether patients with TR require surgical intervention for TR during mitral valve replacement. Specifically, we suggest that measures of right atrial transverse diameter, right ventricular end-diastolic diameter, and pulmonary artery pressure can be used as indicators to determine the need for surgical intervention. We will further do the follow-up to estimate the operative results using this method.

## Abbreviations

TR: tricuspid regurgitation; ACC/AHA: American College of Cardiology/American Heart Association; ESC: European Society of Cardiology; SD: standard deviation.

## Competing interests

The authors declare that they have no competing interests.

## Authors' contributions

Jigang HE: study concepts, study design, definition of intellectual content, literature research, clinical studies, data acquisition, data analysis, statistical analysis, manuscript preparation, manuscript editing; Zhenya SHEN: guarantor of integrity of the entire study, study concepts, study design, manuscript review; Yunsheng YU: clinical studies, data acquisition, data analysis, statistical analysis; Haoyue HUANG: clinical studies, data acquisition, data analysis, statistical analysis; Wenxue YE: clinical studies, data acquisition, data analysis, statistical analysis; Yinglong DING: clinical studies, data acquisition, data analysis, statistical analysis

Shaolei YANG: clinical studies, data acquisition, data analysis, statistical analysis
